# Corrosion and degradation behavior of MCSTE-Processed AZ31 magnesium alloy

**DOI:** 10.1038/s41598-025-88161-7

**Published:** 2025-02-03

**Authors:** Khaled B. Abdelfattah, Marwa A. Abbas, Waleed H. El-Garaihy, Adel M. A. Mohamed, Hanadi G. Salem

**Affiliations:** 1 Metallurgical and Materials Engineering Department, Faculty of Petroleum and Mining Engineering, Suez University, Suez, 43512 Egypt; 2 Department of Mechanical Engineering, College of Engineering, Qassim University, 52571 Buraidah, Saudi Arabia; 3 Mechanical Engineering Department, Faculty of Engineering, Suez Canal University, Ismailia, 41522 Egypt; 4 Mechanical Engineering Department, The American University in Cairo, Cairo, 11835 Egypt

**Keywords:** MCSTE; Biodegradable AZ31 alloy; Corrosion behavior; Microstructure evolution, Engineering, Materials science

## Abstract

This study aims to investigate the impact of multi-channel spiral twist extrusion (MCSTE) on the corrosion and degradation properties of biodegradable AZ31 (Mg-3Al-1Zn, wt.%) magnesium alloy. Square AZ31 billets were processed using route C-MCSTE (with a 180° rotation between passes) at 250°C and with a ram speed of 10 mm/min for up to 8 passes. The extrusion process was conducted via dies with twist angles of 30° and 40°. The microstructural changes and grain size distribution in the alloy were determined with a Scanning Electron Microscopy equipped with Electron Backscatter Diffraction. Electrochemical tests were conducted in a simulated body fluid to model the environment in which medical implants operate. The mechanical properties of the alloy were tested before and after processing using compression tests. The billets processed with a 30° twist angle demonstrated superior mechanical and corrosion resistance compared to those processed with a 40° die. A 66% reduction in grain size was found in billets processed for 4 passes using the 30°-die as compared to the as-annealed condition. Billets processed for 4 and 8 passes showed ultimate compressive strength improvements of 23% and 31%, respectively compared to the as-annealed condition. The 8-pass processed sample using the 30° twist ring die showed 76% improvement in the corrosion rate compared to the as-annealed state. Furthermore, billets processed for 4 passes showed corrosion resistance and ultimate compressive strength improvements of 108% and 23%; respectively compared to the as-annealed condition. These findings imply that the developed MCSTE process can be adopted for industrial use, especially in the manufacturing of biodegradable magnesium alloys for medical implants.

## Introduction

Magnesium (Mg) alloys are among the lightest alloys with a great strength-to-weight ratio, making them ideal for weight-saving in structural applications. Mg alloys are also strong candidates for usage as biodegradable implants due to their density (1.8–2.1 g/cm^3^) and modulus of elasticity (41–45 GPa), both of which are closest to that of human bone compared to other materials. On the other hand, due to their hexagonal close-packed (HCP) crystal structure, which limits active slip systems at room temperature, Mg alloys possess poor ductility and formability^1–5^. To avoid these constraints, Mg-based alloys are commonly found in numerous series (AZ, AM, AXE, ZE, ZK, etc.) with variations of soluble elements. The AZ (Mg–Al-Zn) series, interestingly, possesses better strength and formability at room temperature due to the presence of solute elements like Aluminum (Al) and Zinc (Zn). Among these, the AZ31 alloy is one of the most extensively used due to its widespread commercial availability^6^. Furthermore, the addition of Al to Mg alloys enhances tensile strength and corrosion resistance, while Zn contributes to precipitation hardening and solid solution strengthening^7^. The primary challenge with these alloys that affects their usage in biomedical applications is their rapid deterioration and corrosion in biological environments which affects the mechanical reliability of the alloy during usage^8^.

Biocompatibility, biodegradability, mechanical strength, and stability are major limiting factors in the choice of implant materials^9,10^. One of the main benefits of using a biodegradable material is the associated cost and time savings emerging from eliminating the need for extra treatments or operations to extract the implant from the patient’s body. An added benefit is the painless implant removal, since the implants degrade over time and are eventually replaced by host tissue. Due to their adequate mechanical properties such as strength, ductility, flexibility and relatively low density, compared to that of the bones, metals have been a common biodegradable material for usage in bone repair and auxiliary treatment applications^11,12^. The hazardous byproducts that are created when implants deteriorate are the primary issue with biodegradable metals. However, for Mg alloys, this is no issue, since the adult body stores about 30 g of Mg in both muscle and bone tissue which can be considered medically advantageous^13^. The significance of magnesium to the body is a result of the fact that it is a bivalent ion employed in some metabolic processes as well as to produce apatite in the bone matrix^3^. The main drawback of using Mg alloys as biodegradable implants is the high rate of disintegration in physiological and aquatic conditions. Such problems can be eliminated by tailoring Mg alloys properties through techniques that allow control over the alloy’s grain size and texture during processing. One of these techniques is severe plastic deformation (SPD)^14^.

SPD imposes large amounts of strain with almost no shape or dimension changes during deformation. This results in measurable grain refinement compared to conventional deformation techniques^15,16^. Due to the limited deformability of most materials, restoration mechanisms such as dynamic recovery or dynamic recrystallization may be inevitable during SPD^17^. Restoration mechanisms result in crystalline imperfections re-arrangement and/or annihilation and hence more strain can be tolerated within the material. Using SPD to generate ultrafine-grained (UFG) materials can improve the mechanical properties and regulate the corrosion rate. Therefore, this is especially important for developing functional biodegradable magnesium materials^18^.

SPD techniques are numerous. Among these techniques are equal channel angular pressing (ECAP)^19–22^, twist extrusion (TE)^23–25^, high pressure torsion (HPT)^26–28^, and accumulative roll-bonding (ARB)^29–31^. TE is recognized as a consistent and efficient method for producing UFG metallic materials, which is apparent from the mechanical and corrosion properties of the processed materials. TE involves a high strain gradient and stretching and mixing of metal particles in a vortex-like flow which results in the stabilization of the microstructure and properties of the material^32^. Different routes and/or assembly alterations can be combined to obtain specific properties for various applications. Such flexibility may not be present in many other SPD techniques^33,34^. A modified multi-channel spiral twist extrusion (MCSTE) technique has been recently developed to overcome the limitations of the conventional TE die set up^35^.

**Fig. 1:**
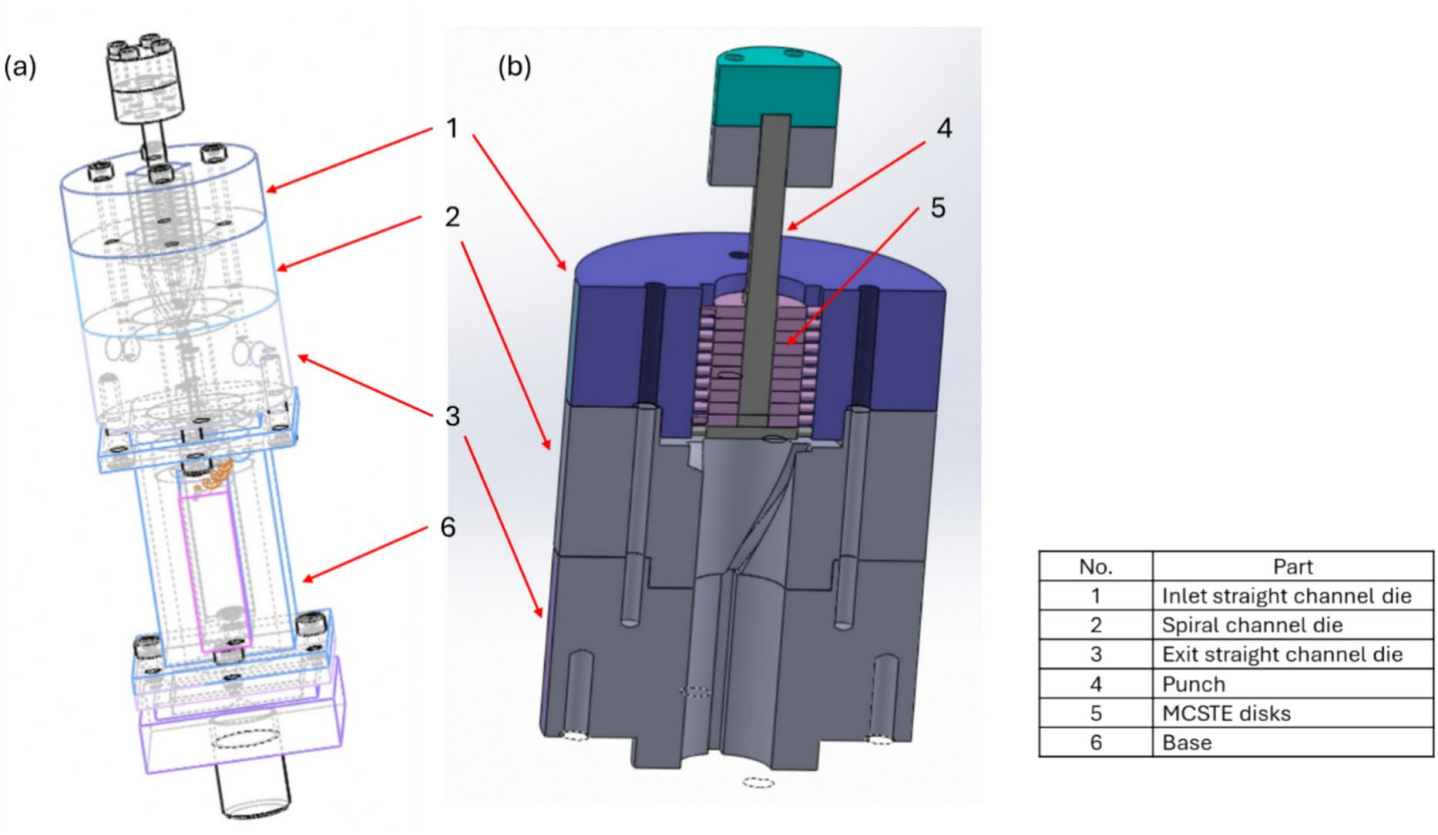
(**a**) A schematic of MCSTE die, (**b**) Sectional view for MCSTE die^39^.

Previous studies had used MCSTE for up to four passes to produce a significant strain buildup^36^. The billet is fixed while surrounded completely by disks and pressed to move through the die portion undergoing twist extrusion as shown in Fig. 1^37^. MCSTE can be used to handle artificial green compact powders, ingot materials (IM), or compact incompletely sintered powders. Hence, the major benefit of the modified set up is the feasibility of scaling it up for industrial production using various twist slope angles (β) enhancing the billets properties for different applications. In addition, different routes can be applied to the billets during processing^36–38^. The most common routes are route A and C. Route A means zero rotation of the billet. In route C, the billet is rotated 180° without flipping the top and the bottom after each pass^39^. The crystallographic texture and its intensity are affected differently by various route types, which in turn alters the billet properties^40^.

In this article, a biodegradable AZ31 (Mg–Al-Zn) alloy was selected for processing by MCSTE with optimized processing conditions. Characterization tests evaluating the microstructural, mechanical and corrosion/degradation properties were carried out to assess the alloy’s suitability for usage as a temporary implant material in bone repair applications. To validate the effects of MCSTE processing, a reference as-annealed (AA) billet was tested as a reference. Billets were processed through route C. Scanning Electron Microscopy (SEM) equipped with Electron Backscattered Diffraction (EBSD) and X-Ray Diffraction (XRD) were used to examine the development of the crystallographic texture, the microstructure as well as the grain sizes as a function of the MCSTE twisting angle and number of passes. To simulate the environment in which the medical implants will be used, electrochemical studies were carried out using the ringer lactate solution. To experimentally assess the samples corrosion properties, electrochemical impedance spectroscopy (EIS), potentiodynamic polarization, open circuit potential (OCP), and weight loss tests were carried out. The mechanical properties of the processed materials were also evaluated using a compression test.

## Materials and methods

Commercial wrought AZ31 alloy with a chemical composition of 2.8 wt.% Al, 0.4 wt.% Zn and balance Mg (China Jingan Chemicals & Alloy Limited, Shanghai, China) was used and sectioned into square billets with the same dimensions of 10*10*40 mm. Before processing, all the billets were annealed at 400 ℃ for 16 h and then cooled in the furnace. The MCSTE processing designations are based on the number of processing passes (1, 2, 4 and 8). Two MCSTE dies were used with twisting angles of 30º and 40°, respectively, to investigate the effect of increasing the plastic strain via increasing the die twist angle. For the first die with a twist angle of 30º, the samples were processed at a temperature of 250ºC. However, for the second die with a twist angle of 40º, 250ºC was insufficient due to the high plastic strain, leading to sample fracture during processing. Consequently, the processing temperature was increased to 300ºC. All billets were processed via route C (rotating the sample 180º perpendicular to its longitudinal axis after the subsequent passes) at a constant strain rate of 10 mm/min using universal testing machine; Schenk-Trebel, as shown in Table 1. The full design description of the MCSTE technique was reported by El-Garaihy et al.^35^.

**Table 1:** Condition designations of different MCSTE process parameters.

Condition	Temperature (℃)	Ram speed (mm/min)	MCSTE die angle	Number of passes
4P-30	250	10	30º	4
8P-30	250	10	30º	8
1P-40	300	10	40º	1
2P-40	300	10	40º	2

The microstructures of the billets were evaluated on longitudinal cross sections cut from the center of the billets. The samples were cold mounted in epoxy and ground using silicon-carbide sandpaper, followed by polishing using alumina suspension (1 µm then 0.5 µm particle sizes). Etching was done using a solution of 10 mL acetic acid, 6 g picric acid and 100 mL ethanol (95%) for 15s according to ASTM E407-07. Microstructural evolution under different processing conditions was examined using EBSD data collected from the top surface extrusion direction (ED) plan in 80 nm increments with the HKL Flamenco Channel 5 software (Hitachi, Ltd., Tokyo, Japan). The SEM was operated at a current of 1.5 nA and a voltage of 15 kV. The contouring parameters were based on more than 10-pixel, data clustering of 5 degrees and half-width of 10 degrees. Energy-Dispersive X-ray Spectroscopy (EDS) was used for elemental analysis. Structural analysis was conducted using X-ray diffraction (XRD, JEOL JDX-8030) with a scan range of 20˚ to 80˚ at a rate of 2˚min^-1^. An accelerating voltage of 40 kV and current of 30 mA were used with Cu Kα (λ = 1.5406 Å) radiation.

Four electrochemical tests were performed to investigate the AZ31 alloy’s corrosion behavior. Most data were recorded using a Metrohm Autolab corrosion device (Herisau, Switzerland). Firstly, open circuit potential (OCP) tests were conducted. The second set of tests included linear and cyclic (potentiodynamic) polarization with a potential range of ± 250 mV against OCP and a scan rate of 0.17 mVs^−1^ to guarantee steady-state performance. The third test involved Electrochemical Impedance Spectroscopy (EIS) experiments at OCP, using sinusoidal voltages within a potential frame of ± 10 mV and frequencies ranging from 100 to 1000 kHz. The experimental set-up consisted of a 3-electrode flat corrosion cell with a counter electrode of platinum mesh, a reference electrode of saturated calomel electrode (SCE) and working electrodes of the prepared billets. Room-temperature ringer lactate served as the corroding agent for all electrochemical tests. The fourth test was an immersion test for 30 days to investigate weight loss in a ringer lactate solution. For the immersion test, the billets were sectioned into 10*10 mm squares with thicknesses less than 5 mm. After the immersion test, AZ31 billets were cleaned using 180g/L chromic acid at room temperature for roughly 10 min to eliminate the corrosion products then rinsed with distilled water and let to air dry. Several measurements are taken until the billet weight value stabilizes.

The billets mechanical properties were assessed via compression testing. Compressive tests were conducted at room temperature according to ASTM E9 using Instron universal testing machine at a strain rate of 0.01 min^-1^.

## Results and discussion

### Microstructure and phase identification

**Fig. 2:**
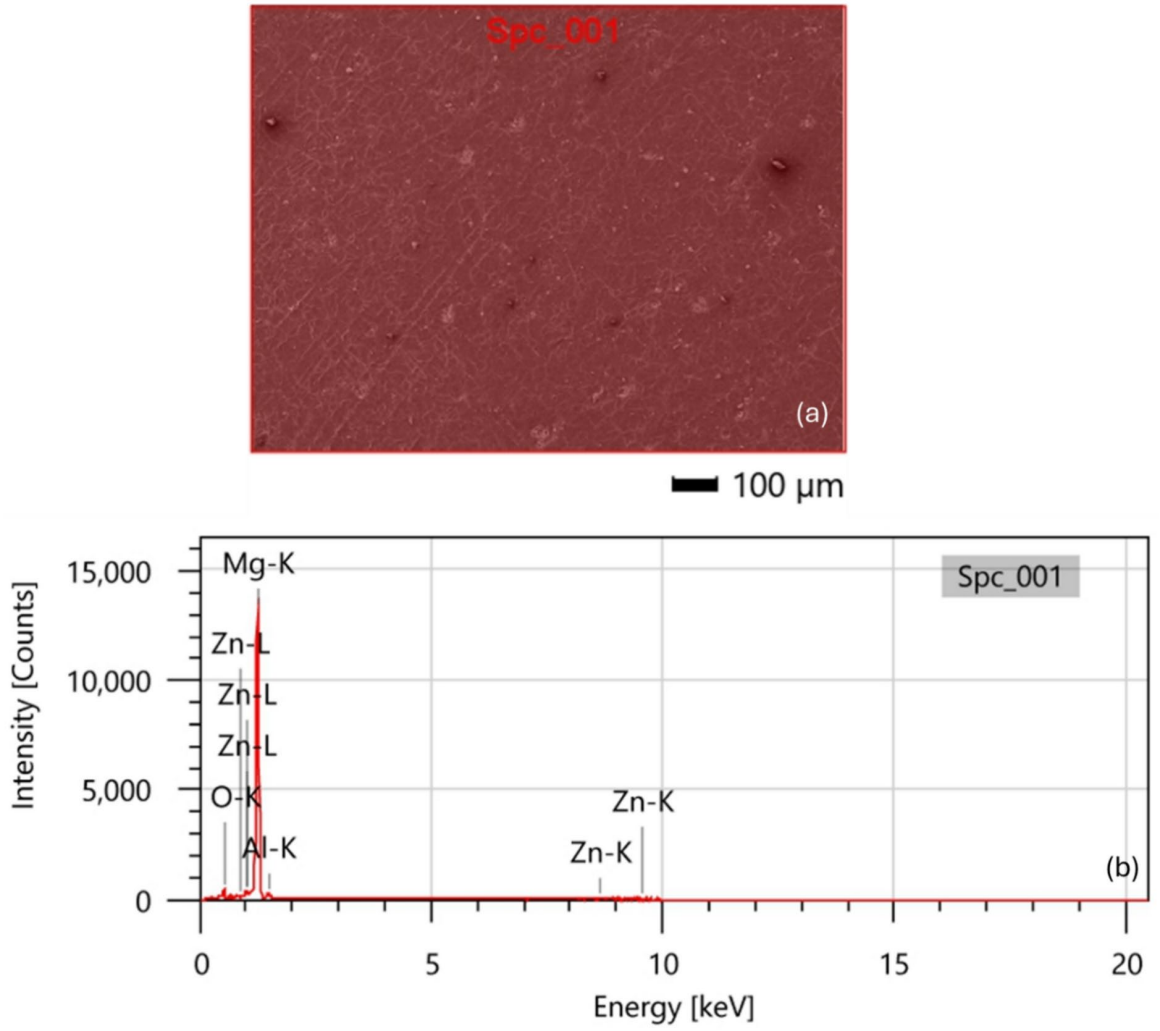
(**a**) SEM surface morphology and (**b**) EDS spectrum analysis for AZ31 alloy.

The EDS spectrum analysis, presented in Fig. 2, confirms that the investigated AZ31 alloy contains 2.75 wt.% Al and 0.38 wt.% Zn. The presence of Mg and Al elements is assumed to be associated with the existence of a precipitated phase, most likely Mg_17_Al_12_ as investigated via the XRD analysis discussed later^41^.

**Fig. 3:**
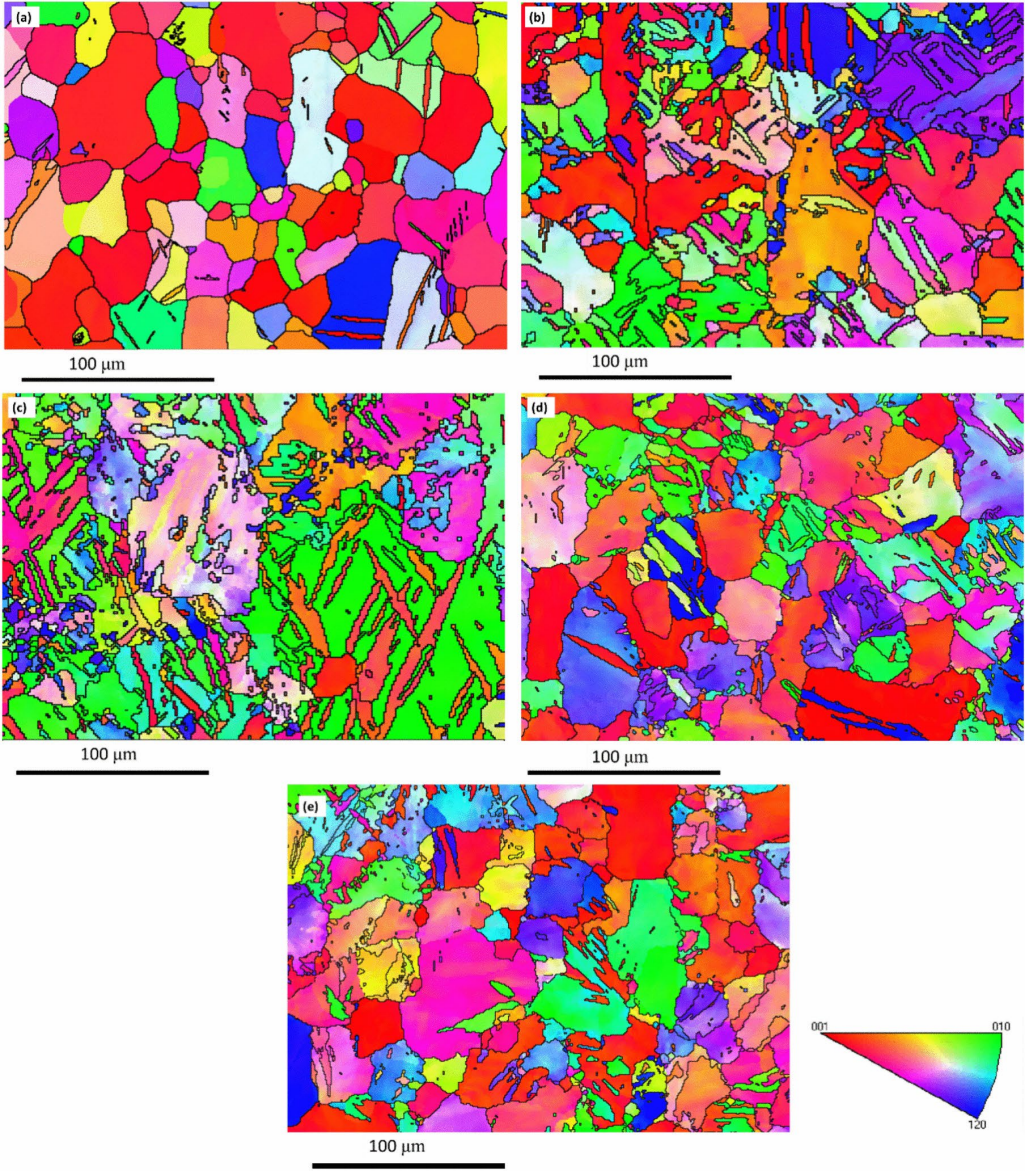
IPF coloring maps of the AZ31 billets relative to the extrusion direction after MCSTE processing at conditions (**a**) AA, (**b**) 4P-30°, (**c**) 8P-30°, (**d**) 1P-40° and (**e**) 2P-40°.

**Fig. 4:**
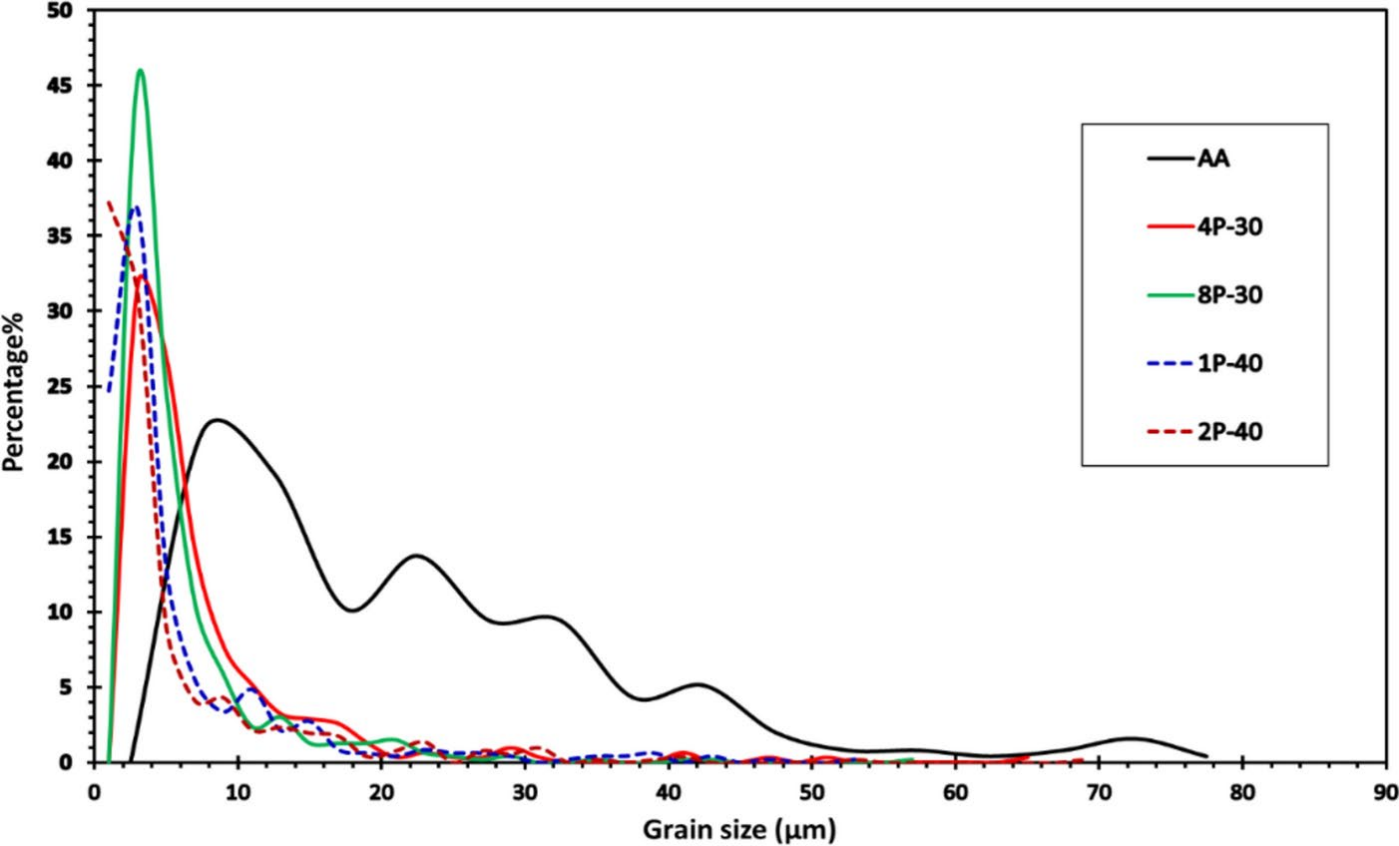
Grain size distribution of the AZ31 billets after AA and MCSTE processing.

**Fig. 5:**
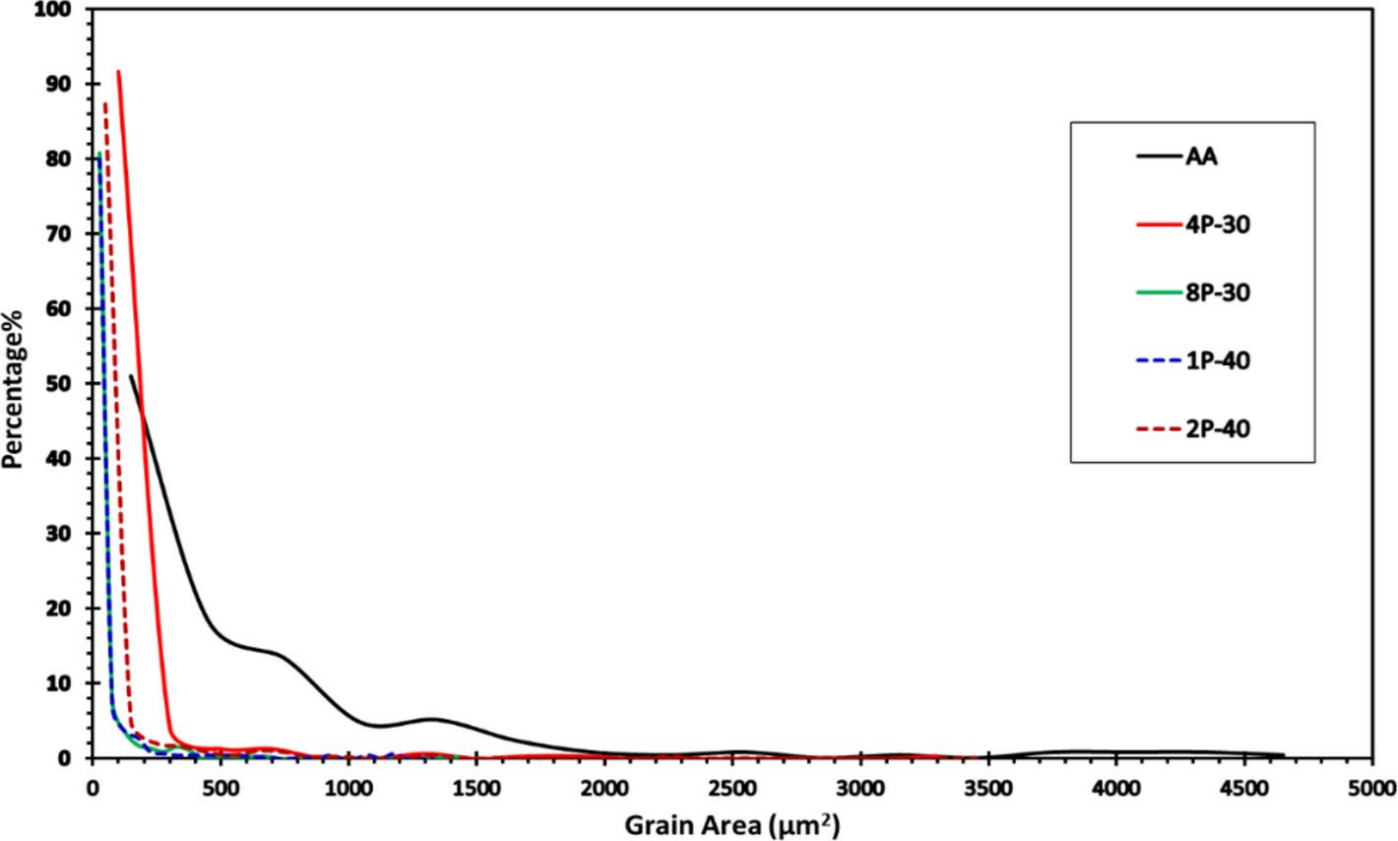
Relative frequency of grain area distribution of AZ31 billets before and after MCSTE processing.

**Fig. 6:**
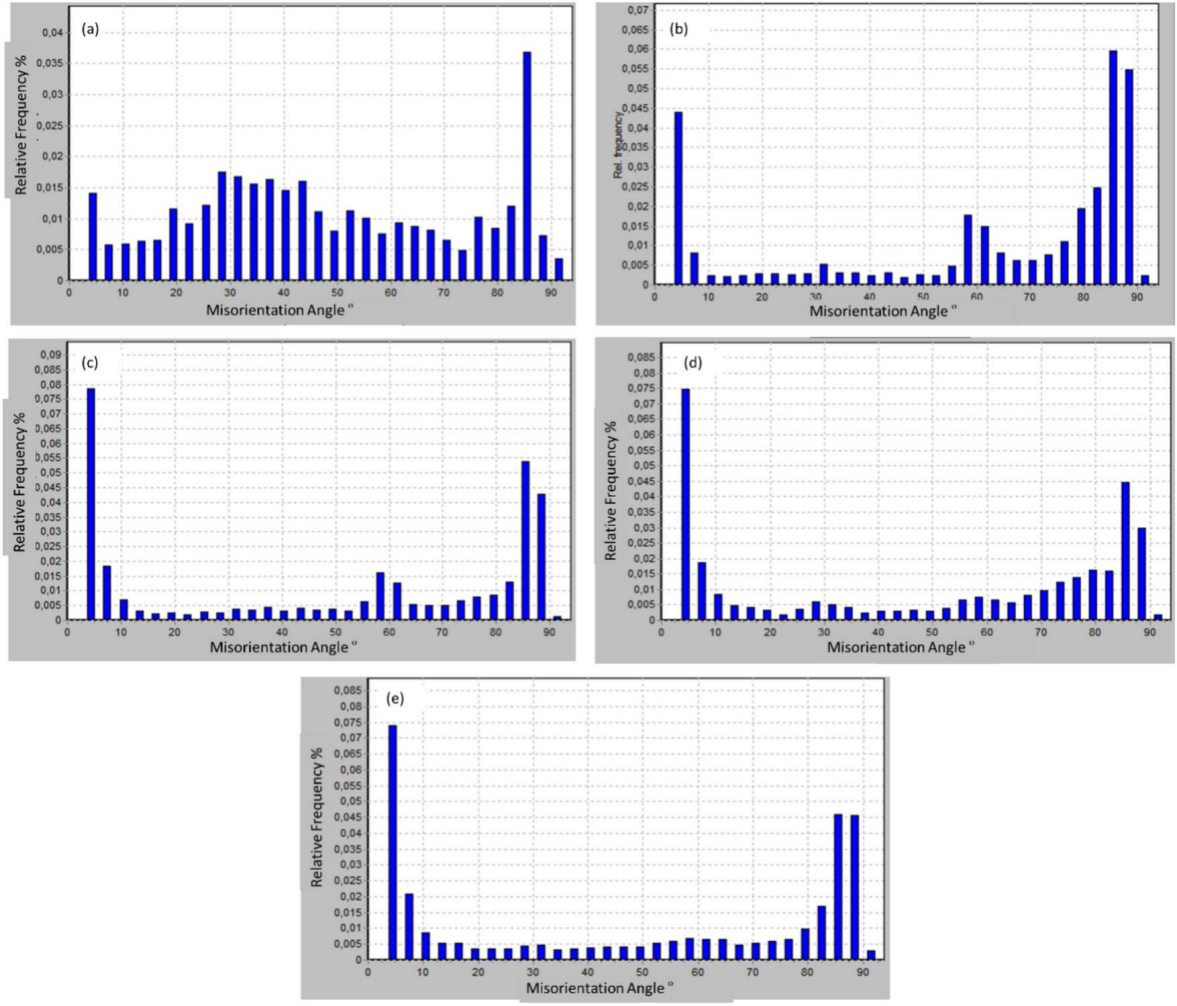
Misorientation angle distribution (a) AA, (b) 4P-30°, (c) 8P-30°, (d) 1P-40° and (e) 2P-40°.

The Inverse Pole Figure (IPF) colored maps relative to the extrusion direction of the AA billet compared to the processed billets for the four conditions (Table 1) are shown in Fig. 3. Table 2 displays the minimum, maximum, and average grain sizes coupled with the distributions’ standard deviations of the four MSCHT processed conditions. A comparison between grain size distributions of the AZ31 billets is shown in Fig. 4, while that of grain area distributions is shown in Fig. 5. Furthermore, misorientation angle distribution is shown in Fig. 6.

**Table 2:** Grain size data for the AA and processed AZ31 billets. All units are in µm.

	AA	4P-30	8P-30	1P-40	2P-40
Min	5.05	2.52	2.26	1.35	1.17
Max	76.90	64.33	57.23	52.13	68.46
Average	22.18	7.60	6.25	5.96	5.41
Standard Deviation	14.78	7.37	5.95	7.48	7.51

Post annealing, the AA AZ31 billets revealed a combination of fine grains (> 5.1 µm) and equiaxed coarse grains (< 76.9 µm). The average grain size was 22.2 µm and the average grain area was 557 µm^2^ (Figs. 3a and 5). MCSTE processing resulted in significant grain refinement as shown in Table 2. Processing via 4P-30° produced non-uniform grain structure ranging between 2.5 to 64.3 µm, with an average grain size of 7.6 µm, which represent 66% grain size reduction compared to that of its AA counterpart after processing. In addition, processing 4P-30° via MCSTE resulted in reducing the average grain area by 84% compared to the AA condition. The 8P-30° billet produced improvement in the uniformity of grain refinement ranging between 2.26 to 57.2 µm with an average value of 6.2 µm. This demonstrates a grain refinement of about 72% compared to the AA billet. Furthermore, the average grain area of 8P-30 was reduced by nearly 90% compared to the AA billet. The 1P-40 billet developed grain sizes ranging from 1.4-to-52.1 µm with an average value of 5.96 µm. This indicates reductions of about 73% and 87% in the average grain size and grain area, respectively, compared to the AA billet as shown in Figs. 4 and 5. The 2P-40° billet’s grain sizes ranged from 1.2 to 68.5 µm with an average of 5.4 µm, producing grain refinement of about 76% compared to the AA billet. Furthermore, processing through 2P-40° yielded a reduction of 88% in the average grain area compared to the AA condition, which indicates that the refinement effect of 2P-40° is like that of the 8P-30° condition, as seen in Fig. 5. Other studies on AZ31 processed through ECAP have similar findings with average grain size of annealed samples approximately 50–20 μm, and average grain size of the ECAPed samples reduced to 10–3 μm^42^. This indicates that MCSTE is comparable to ECAP in refining the grain structure of the AZ31 alloy.

The structure uniformity increased with increasing the number of passes up to 8P-30°, yielding higher fine-grain percentages of up to 45% (Fig. 3c). On the other hand, the effect of the die twist angle can be clearly; AZ31 samples processed through 1P-40° showed an ultrafine grained structure (UFG, less than 1 μm) percentage of 25%, whereas the percentage of fine grains increased to 61.5%. An increase in structural uniformity was observed in the AZ31 alloy processed through 2P-40°; the percentage of the UFG structure increased to 37% whereas the percentage of the fine-grain structure increased up to 68% (Fig. 4). This confirms that increasing the twist angle increases grain refinement due to the higher strain accumulation, as previously reported by^39^. It can also be concluded that grain refinement with increasing the number of MCSTE twisting passes, is attributed to the increased cumulative effective strain which extends form the peripheries of the die-billet interface toward the center,^35–37^.

Regarding the misorientation angles, the band contrast map (Fig. 6a) shows the dominance of HAGB structures for the AA condition, which indicates full recrystallization. Processing through multiple passes of MCSTE resulted in the continuous dynamic recrystallization (cDRX)^36^. During cDRX, the misorientation of low-angle subgrain boundaries increases due to deformation till they develop to high-angle grain boundaries^36^. The misorientation angles distribution in Fig. 6b and c shows an appreciable fraction of LAGBs, which supports the claim that cDRX was the formation mechanism behind the new grains for AZ31 alloy processed through 4 and 8 passes, respectively using the 30º-die. A similar trend was revealed for the AZ31 billets processed through 1 and 2 passes using the 40º-die as shown in Fig. 6d and e, respectively.

**Fig. 7:**
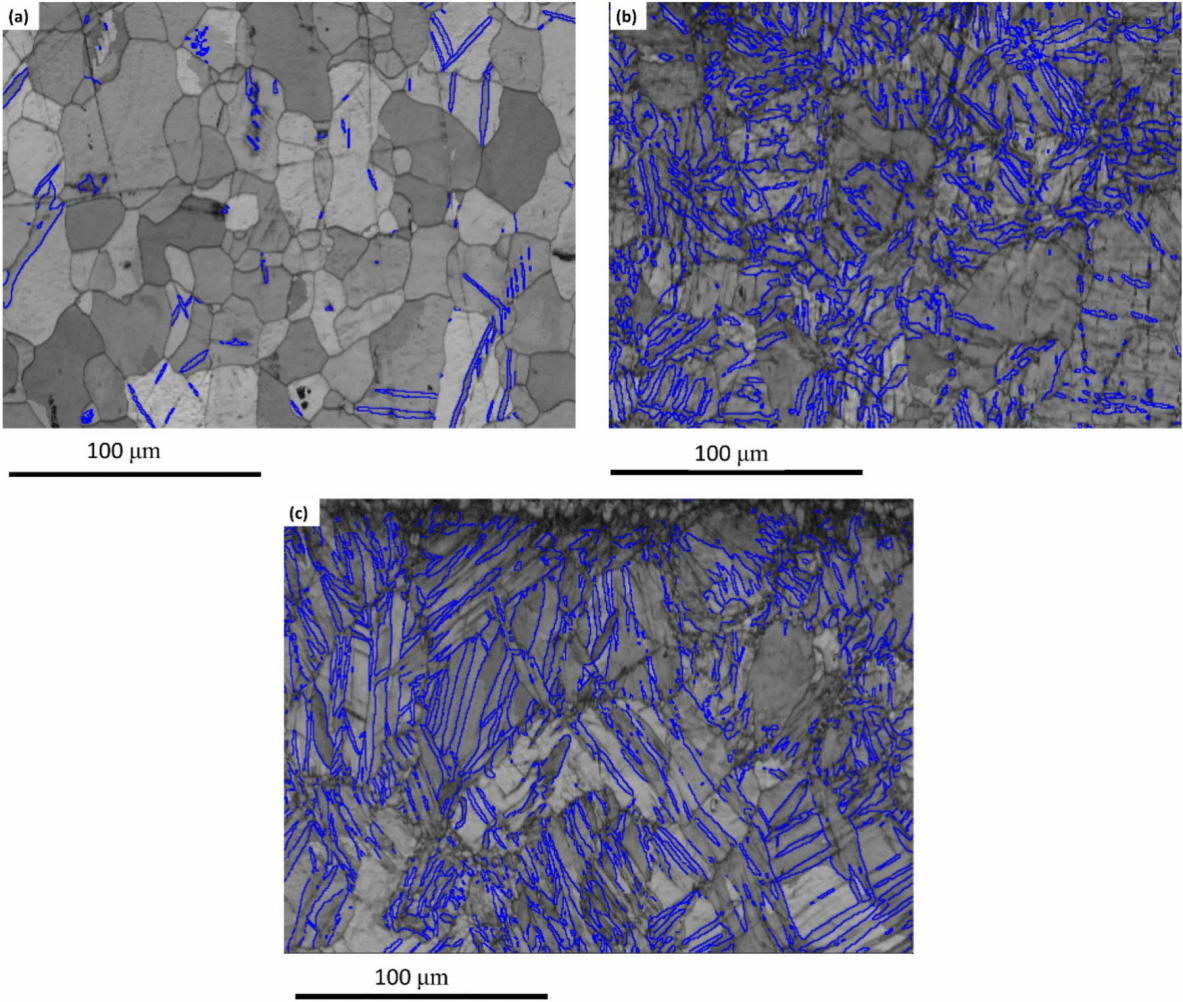
EBSD grain boundary maps (˂2^–^1^–^10˃, at angle 86º) of AZ31 samples with different processing conditions (**a**) AA, (**b**) 4P-30°, (**c**) 8P-30°, blue lines indicate twin boundaries.

Figure 7 shows EBSD micrographs of AZ31 alloy billets cut parallel to the extrusion direction for the increasing number of passes vis MCSTE twisting angle of 30°. The coarse-grained samples (AA) displayed almost twin-free microstructure as the twins were depicted only in a very limited number of sites as shown in Fig. 7a. Processing via MCSTE as a function of increasing the number of passes resulted in a significant increase in twin boundaries within the deformed grains as shown in Figs. 7b and c, for the 4P and 8P passes, respectively. Furthermore, increasing the number of MCSTE passes produced finer twin boundaries as depicted in Fig. 7c (8-passes) compared to Fig. 7b (4-passes), which is consistent with the findings of Lou et al.^43^.

It has been reported that SPD processing can produce two deformation mechanisms in Mg-alloys: twin boundaries created by dynamic recrystallization (DRX) and coarse grain (CG) α-Mg separated by deformation networks with UFGs nucleated along the grain. These structures shown in Fig. 3 can result from processing-induced grain refinement^44–46^. The fine grains that nucleated at the boundaries of coarser grains confirm that deformation and grain refinement began at the grain boundaries (GBs) with the increase in number of passes from 4 to 8P-30° (Fig. 3c). The lattice imperfections generated by both SPD and DRX are expected to contribute substantially to the decrease in the grain size of the AZ31 alloy^47–50^.

**Fig. 8:**
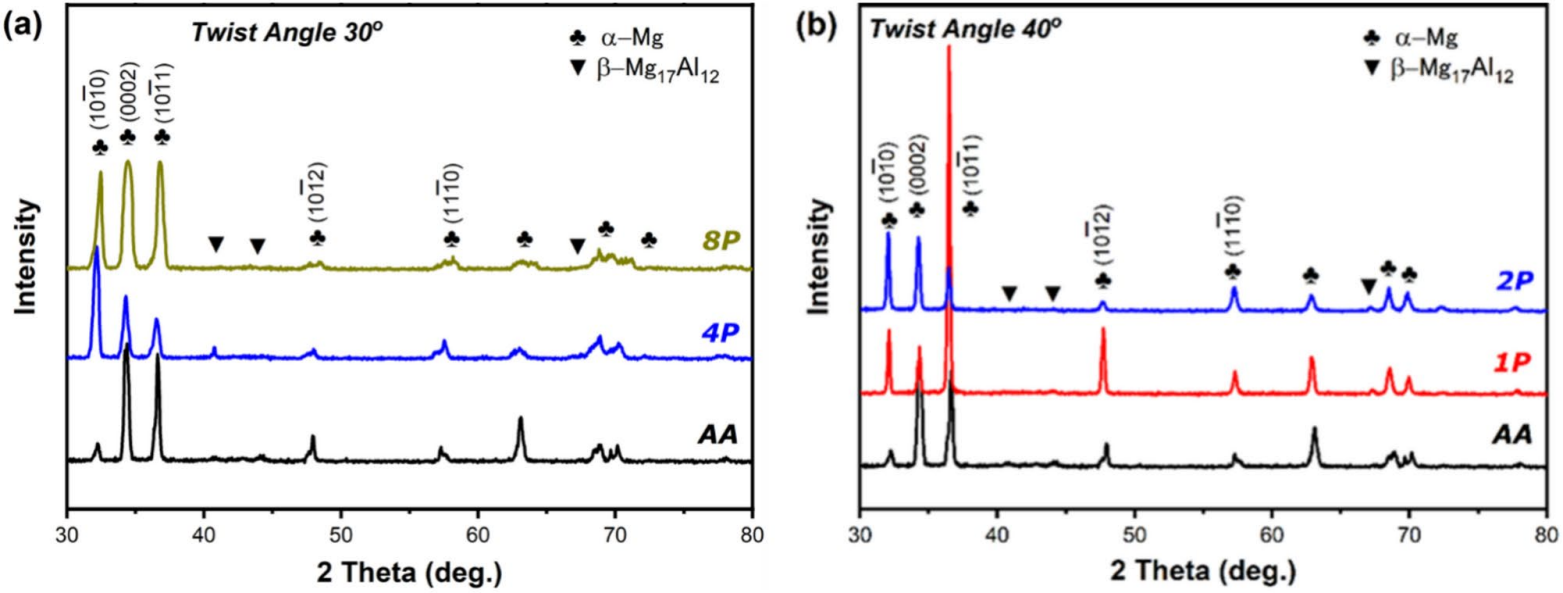
XRD of the of AA and MCSTC-processed AZ31 alloy at (**a**) 30° and (**b**) 40° twist angles.

Figure 8a and b shows the XRD pattern of the AA and processed billets via increasing number of passes and twist angles. The intensities are characteristic of a solid solution of α-Mg and intermetallic β- Mg_17_Al_12_. After annealing the β- Mg_17_Al_12_ phase became hard to detect due to its dissolution in the matrix^51^. Strong (0002) peaks observed in the billets processed by MCSTE indicate that processing with either twist angles induced a strong basal texture, which was more pronounced with the 30˚ die. The (1010) and (1011) peaks can also be observed, and their level of intensity increases with the number of MCSTE passes. As the alloy underwent an increasing number of passes via 30° twist die, the diffraction peaks progressively broadened reflecting the formation of ultrafine structures, the increased intensity of strain and the development of higher dislocation densities and twin boundaries. This phenomenon aligns with previous studies on the relationship between plastic deformation and grain refinement in Mg alloys^52,53^. In general, a basal texture was observed in Mg alloys with a HCP structure because the critical resolved shear stress (CRSSs) of the basal slip is substantially smaller than that of the non-basal slip, which is associated with grain refinement^54^. Notably, after four passes via 30˚ twist die, diffraction peaks representing β-Mg_17_Al_12_ precipitation phases became visible. It is suggested that increasing the induced strain increases the number of localized phase nucleation sites. Similar behavior was observed for AZ31 billets processed through 1P and 2P using the 40°-die, as shown in Fig. 8b.

Several factors contribute to the grain refinement of the AZ31 alloy during the twist extrusion process. Key factors include high strain, twinning, and dynamic recrystallization. Previous studies showed that applying high shear strain results in a combined deformation mechanism that alters dislocation density, initiates twinning in the original grains, and triggers dynamic recrystallization, resulting in the nucleation of fine grains within the initial ones^55,56^. Applying higher strain fractures the Mg_17_Al_12_ phase. After a number of extrusion passes, the Mg_17_Al_12_ phase favors the particle-stimulated nucleation (PSN) mechanism, and pins grain growth as reported by Chen et al.^57^. Subsequent passes crush most particles into smaller ones, and the combination of finer Mg_17_Al_12_ particles and dynamic recrystallization results in a more homogeneous structure, as reported by Alateyah et al.^58^.

### Mechanical properties

**Fig. 9:**
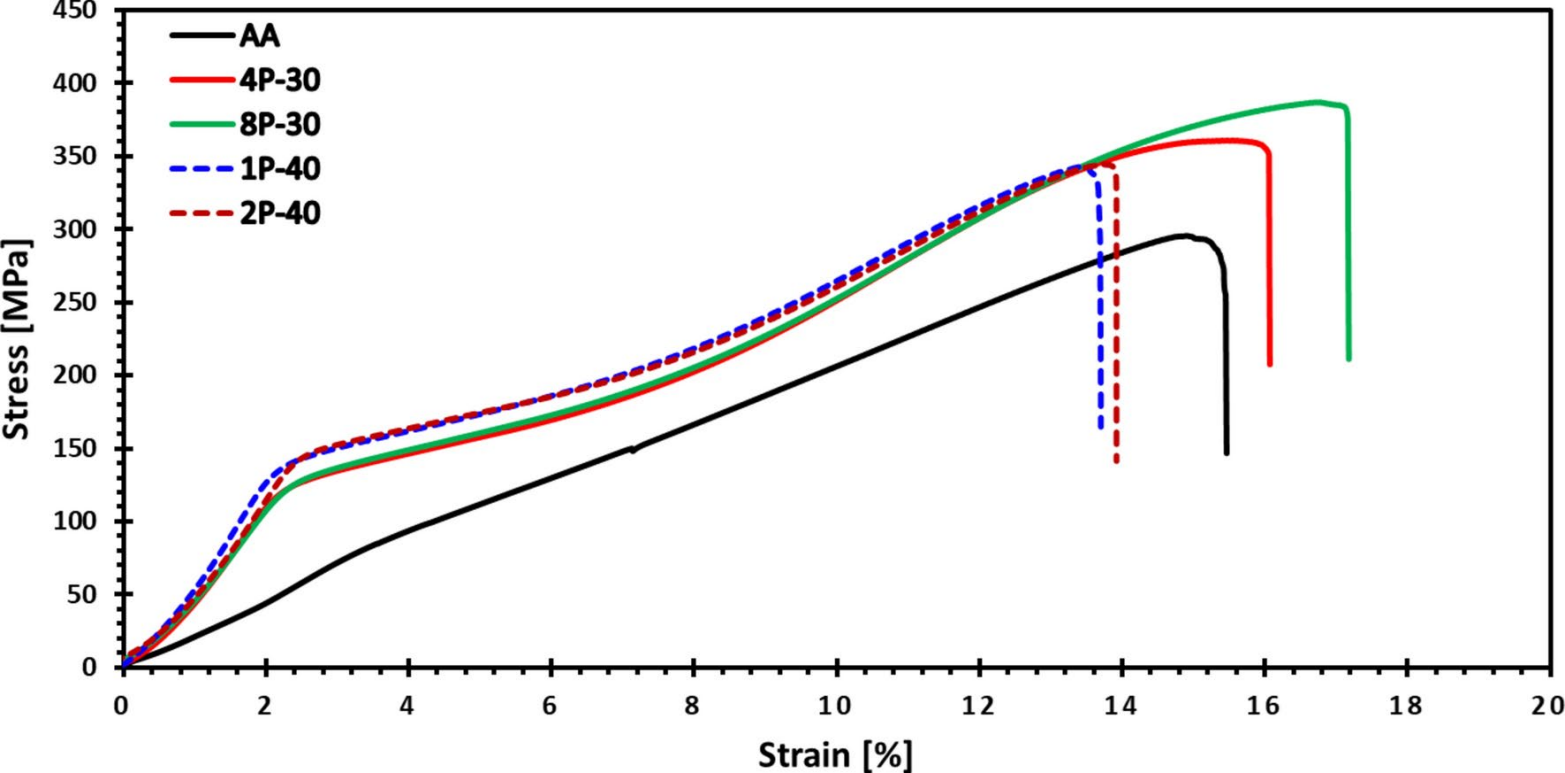
Stress–strain curves of AZ31 billets before and after MCSTE processing.

The Stress–Strain behavior of the AZ31 alloy is displayed in Fig. 9, while the measured yield stress (σ_y_), ultimate compressive strength (σ_u_) and ductility (ɛ_f_) values for the AA and MCSTE conditions are presented in Table 3. It is clear from Fig. 9 that MCSTE processing resulted in a significant increase in σ_y_ compared to the AA condition. Furthermore, there was a noticeable increase in the σ_u_ of processed billets compared to the AA condition, which was coupled with an increase in ductility (ɛ_f_), especially for the processed billets via 30° twisting angle.

**Table 3:** Compressive properties of AZ31 billets before and after MCSTE processing.

Condition	Yield Stress (MPa)	Ultimate Strength (MPa)	Ductility (ɛ%)
AA	92 ± 1.5	295 ± 1.4	15.5 ± 0.4
4P-30	129 ± 1.3	361 ± 1.2	16.1 ± 0.3
8P-30	132 ± 1.4	387 ± 2	17.2 ± 0.6
1P-40	146 ± 2	343 ± 1.7	13.7 ± 0.3
2P-40	147 ± 1.8	343 ± 1.9	13.9 ± 0.4

MCSTE processing via 30° twist channel resulted in an increase of nearly 37% in σ_y_ while processing through the 40° twist channel resulted in an increase of more than 42% in σ_y_ compared to the AA billet. The activation of non-basal slip mechanisms during deformation, may potentially impact the YS of the magnesium alloy^59^. The σ_u_ of the MCSTE billets processed through the 30° twist channel increased by more than 30% compared to the AA billet. In contrast, the billets processed through the 40° twist channel showed a σ_u_ increase of about 17% compared to the AA counterpart. The grain refinement and twin density caused by the accumulation of strain discussed earlier are the dominant means by which the yield and ultimate strength increased. Similar studies on AZ31 processed via ECAP mentioned that the average improvement in σ_y_ and ultimate tensile strength (UTS) of about 25–40% and 15–30%, respectively^60^. This indicates that MCSTE can produce processed billets with superior mechanical properties almost akin to ECAPed ones.

As discussed earlier in Sect. 3.1, MCSTE processing resulted in grain refinement that played an important role in strengthening the AZ31 Mg-based alloy. This is validated by the rise in σ_u_ and is consistent with the Hall-Pitch relationship. The grain refinement after MCSTE processing can be explained by the increase in dislocation density and the homogeneity of the β-Mg_17_Al_12_ intermetallic phase after SPD processing^61^. The relatively smaller increase in mechanical properties of the processed billets through 40° MSCTE angle can be explained by the occurrence of incomplete DRX which led to less refinement of the grains and the precipitated phase and hence a slight reduction in ductility occurred^62^. It has been proven that the absence of slip systems in hcp metals, such as magnesium, causes a significant dependence of strength on grain size^63^. Additionally, the twinning effects caused a higher dislocation density and hence intense lattice strain was the main cause behind the increased toughness of billets processed through the 30-degree channel^64^. However, the slight decrease in ductility at conditions 1P-40 and 2P-40 is suggested to have occurred due to incomplete DRX, which resulted from the high strain accumulation associated with the increased twist angle, as discussed earlier. Furthermore, it is suggested that MCSTE processing via a 40° twist angle caused dislocation generation and multiplication, which raised the dislocation density. These increased density of the dislocations whose motion was impeded at the twin boundaries resulted in the decrease in toughness compared to processing through the 30-dergree channel^65,66^.

As shown in Fig. 9, slight increases in ductility of approximately 4% and 11% were observed for the 4P-30° and 8P-30° billets, respectively, compared to the AA. This increase in ductility was accompanied by notable improvements in both ultimate and yield strengths. The ultimate strength increased by 22% and 31% for the 4P-30° and 8P-30° billets, while the yield strength increased by 40% and 43%, respectively, compared to the AA billet. These results indicate the material’s enhanced ability to withstand load before yielding and fracture, attributed to a refined grain structure and strain hardening effects. Furthermore, strain softening, promoted by the multiple heating cycles during each pass, contributed to this behavior. The cumulative softening effect became more pronounced with additional passes, as increased heating time facilitated recovery, counterbalancing strain hardening. In contrast, for the 40° die, ductility decreased by approximately 11% and 10% for the 1P-40° and 2P-40° billets, respectively, compared to the AA billet. Despite this reduction in ductility, both ultimate and yield strengths still exhibited substantial enhancements of 16% and 60%, respectively, for the 1P-40° and 2P-40° billets, relative to the AA. This demonstrates that strain hardening played a dominant role during processing with the 40° die. The decreased ductility after two passes with the 40° die, compared to the 30° die, underscores the greater strain hardening intensity imposed per pass with the 40° die. These findings agree with the study by Alateyah et al.^67^.

### Electrochemical measurements

**Fig. 10:**
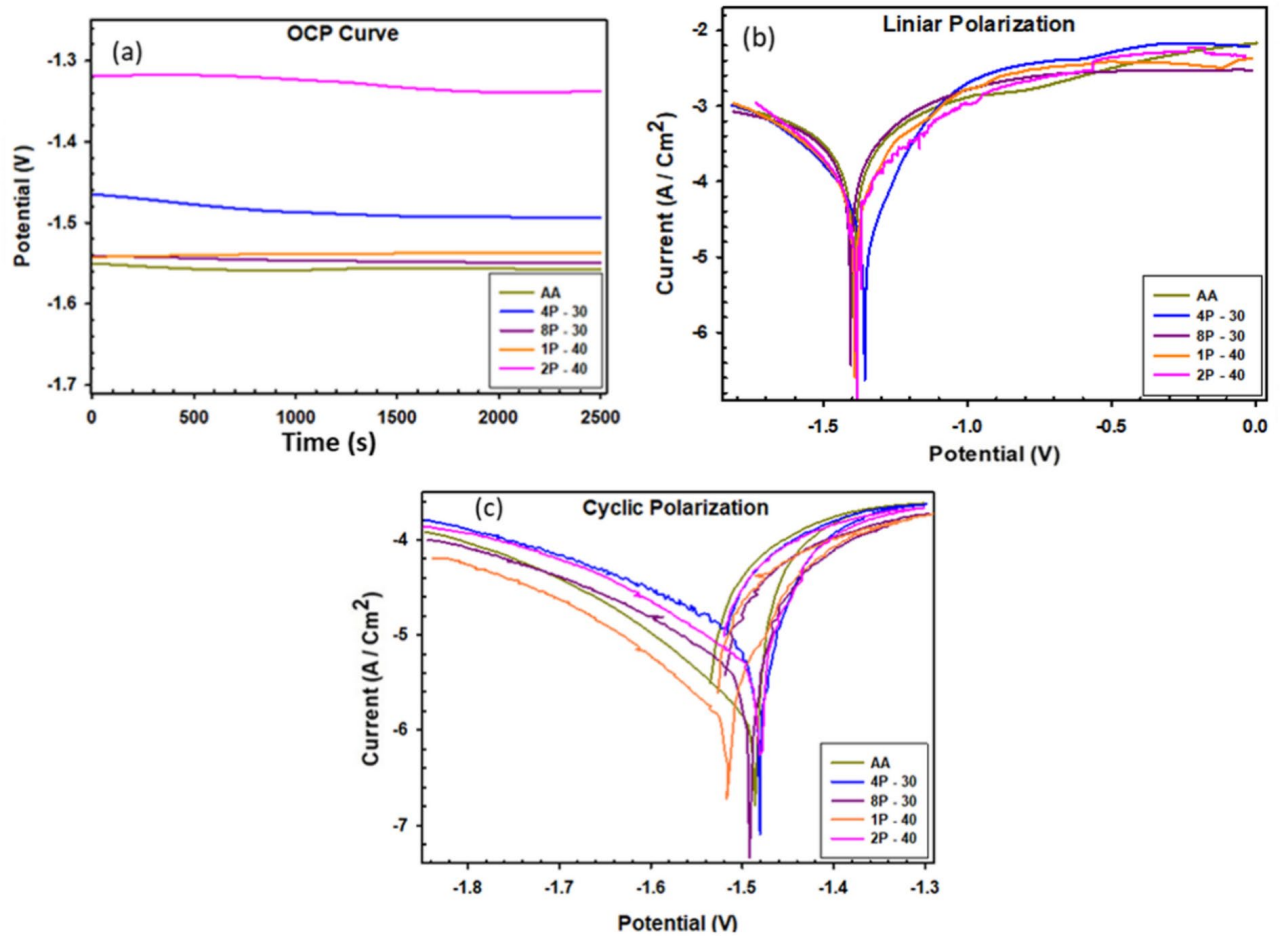
Electrochemical behavior of AZ31 alloy processed through MCSTE: (a) OCP curves, (b) linear polarization curves, and (c) cyclic potentiodynamic polarization plots.

**Fig. 11:**
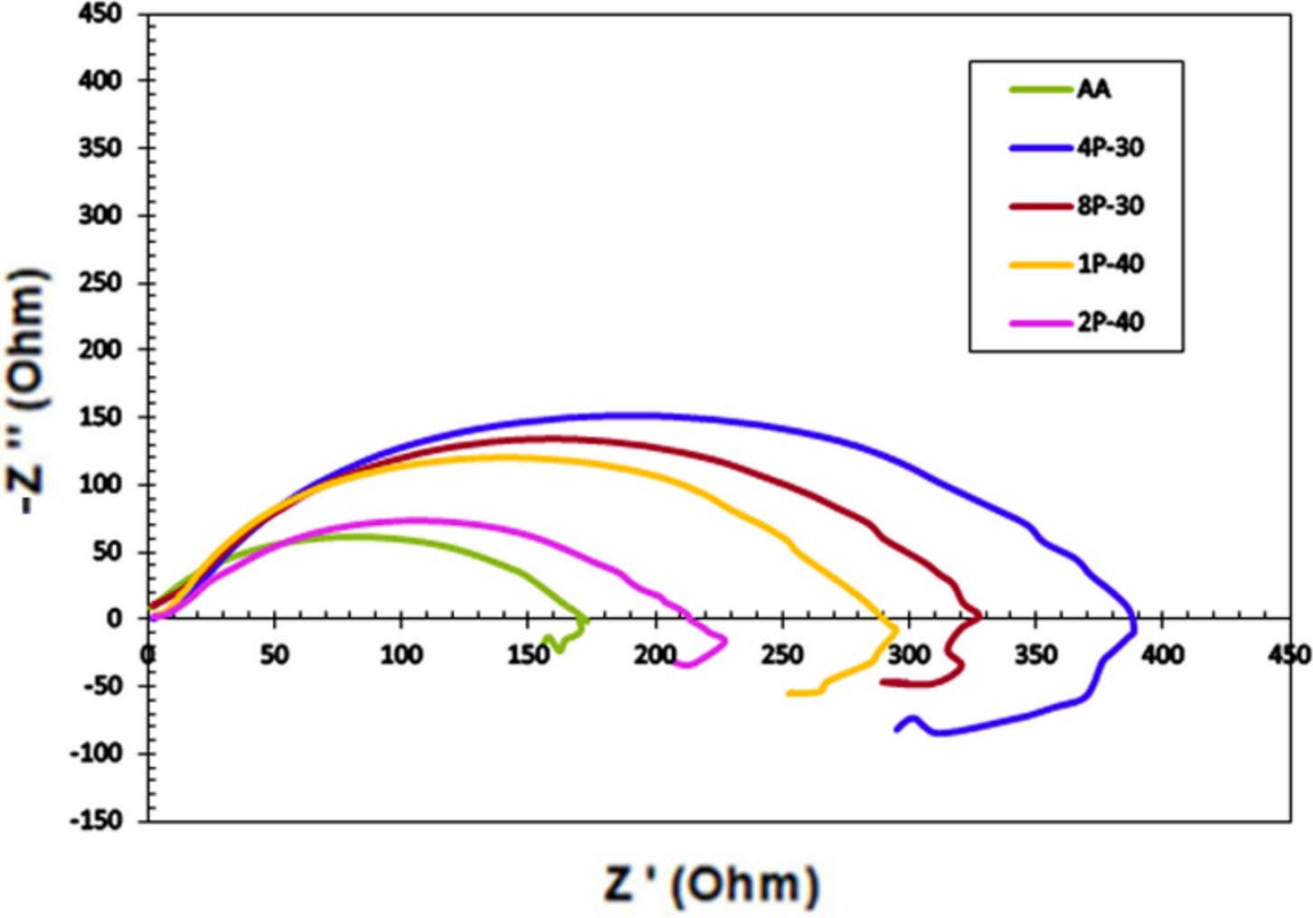
Nyquist plot of AZ31 alloy processed through MCSTE.

Using ringer lactate (pH = 6.5) as a corrosive medium, various electrochemical tests were conducted to examine the corrosion behavior of AZ31 billets before and after MCSTE processing, as seen in Figs. 10 and 11. The OCP of the AA and MCSTE-processed billets are shown in Fig. 10a. The corrosion potential of the AA billets showed a minor drop before stabilizing at -1.507 V, as shown in Fig. 10a. Similarly, the MCSTE-processed billets initially showed minor drops in corrosion potential, followed by a period of steady potential value, and then a significant increase before reaching a constant value (Fig. 10a). All AZ31 billets exhibited trivial differences in potential compared to the AA counterpart, save for the 4P-30 and 2P-40 billets which displayed potentials of − 1.48 and − 1.33 V, respectively. The significant shift in the potential of 4P-30 and 2P-40 conditions to more noble values indicates better corrosion behavior compared to their AA counterpart. The noble OCP shift can be attributed to the formation of a more stable protective layer, which was stimulated by the increased plastic strain (4P-30 and 2P-40) compared to the lower strain conditions.

Figure 10b demonstrates the potentiodynamic polarization curves of all AZ31 conditions. The corrosion current density (Icorr), corrosion potential (Ecorr), anodic and cathodic polarization constants (βa and βc), and the corrosion rate in millimeter penetration per year (mm/y) are presented in Table 4. The corrosion rate can be calculated from the following equation^68^:

1 $$CR= \frac{3.27*icorr*Ew}{1000\rho }$$

where *i*_corr_ is the corrosion current density in µA/cm^2^, E_w_ is the equivalent weight of the corroding metal in grams/equivalent, and p is the density of the corroding metal in g/cm^3^.

**Table 4:** Electrochemical parameters acquired from linear polarization curves of AZ31 alloy.

Condition	βa (V/dec)	βc (V/dec)	E_corr_ (V)	I_corr_ (A/cm^2^)	Corrosion rate (mm/y)
AA	0.22692	0.25379	-1.4202	1.0131 × 10^–04^	2.1766
4P-30	0.1575	0.13081	-1.3624	2.2535 × 10^–05^	0.48417
8P-30	0.11627	0.11455	-1.3851	2.3925 × 10^–05^	0.51404
1P-40	0.21302	0.24479	-1.3912	7.6050 × 10^–05^	1.6339
2P-40	0.2162	0.27853	-1.3776	7.6468 × 10^–05^	1.6429

From 10b, it can be noted that processing through the MCSTE die with a twist angle of 30º caused a notable noble shift in the Icorr, while billets processed through the MCSTE die with a twist angle of 40º showed trivial shifts towards the passive direction. Furthermore, no significant Ecorr shift was observed. For the MCSTE-30º die, processing through 4P caused a significant reduction of 88% in the corrosion rate compared to the AA billets. However, further processing through 8 passes showed an improvement of 76% in the corrosion rate compared to the AA condition. For the MCSTE-40º die, increasing the plastic strain by increasing the helix angle resulted in a notable increase in the corrosion rate compared to the MCSTE-30º die, as shown in Table 4. On the other hand, processing 1 pass and 2 passes using the MCSTE-40º die improved the corrosion rate by 25% and 24% compared to the AA billets. Linear polarization curves confirmed that grain refinement resulting from MCSTE processing led to an improvement in the corrosion rate. The grain refinement caused an associated increase in the grain boundaries area which led to the formation of more stable, more coherent and thicker oxide protective layers (MgO). On the other hand, it can be observed that imposing excessive strain, through further processing using an increased number of passes or by using the MCSTE-40º die for multiple passes, increased the dislocation density and accordingly dislocation entanglement at the grain boundaries. These grain boundaries are very sensitive to the corrosive agent, which commonly causes corrosion to start there. This leads to a higher corrosion rate in samples processed with excess plastic strain, compared to their counter parts with less processing strain, as shown in Table 4, which agrees with earlier studies^69–72^. Studies on ECAPed AZ31 alloy showed that the average reduction in corrosion rate is 65–80% which is very comparable to MCSTE and suggests using the novel processing technique for decreasing corrosion rate of such alloys^73^.

The Cyclic potentiodynamic polarization curves (CPP) are shown in Fig. 10c for both AA and MCSTE processed conditions. All AZ31 billets displayed a complete hysteresis loop which confirms that the AZ31 alloy has the ability to re-passivate after corrosion attacks, which is in agreement with a previous study^74^. The passivation capability of AZ31 alloy can be attributed to the formation of an oxide protective layer which impedes further corrosion after its formation.

**Fig. 12:**
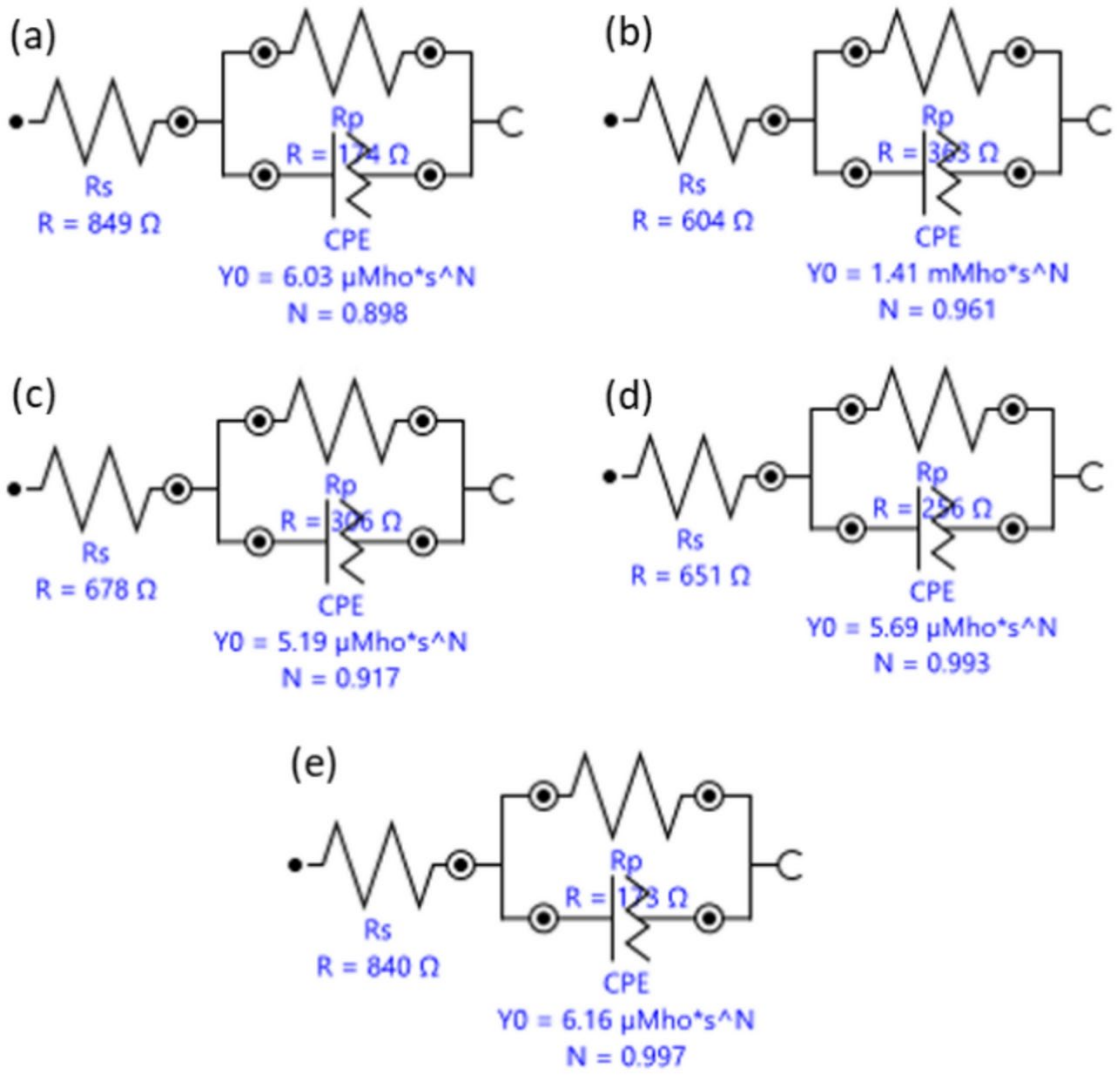
Electrical equivalent circuit for AZ31 samples: (**a**) AA, (**b**) 4P-30º, (**c**) 8P-30º, (**d**) 1P-40º, and (**e**) 2P-40º.

**Fig. 13:**
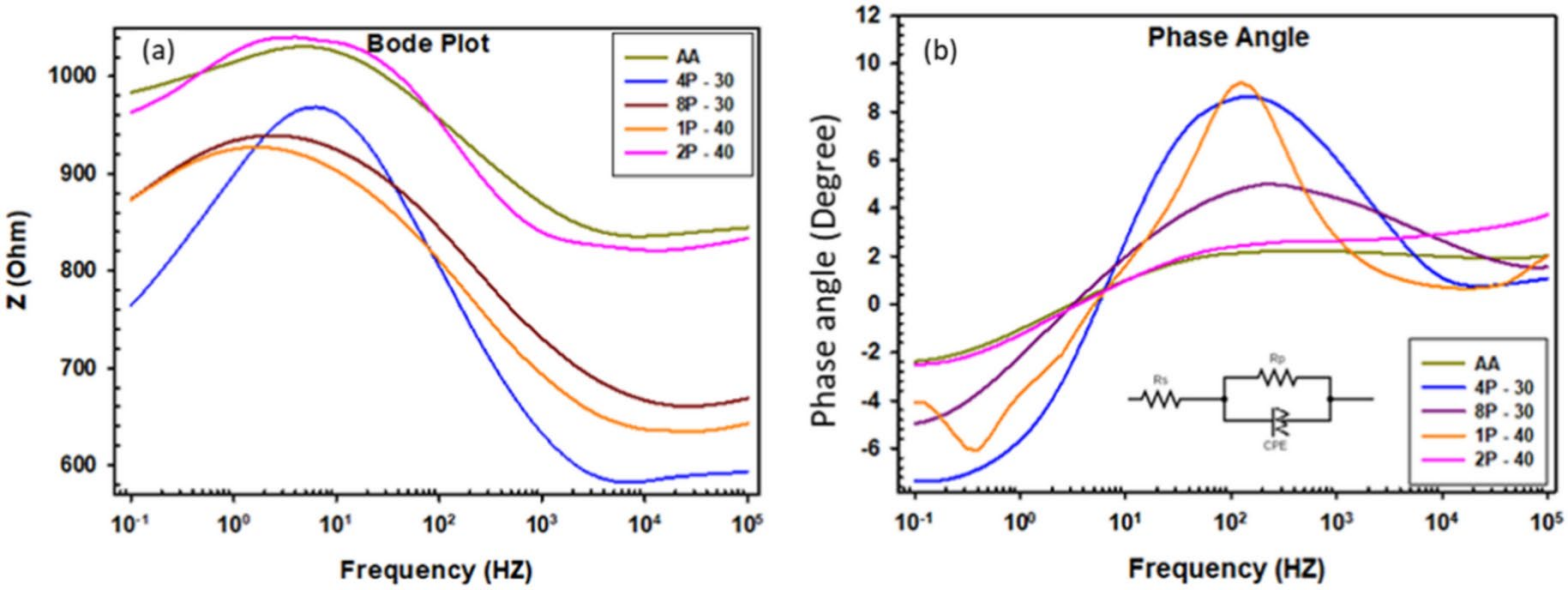
Electrochemical behavior of AZ31 alloy processed through MCSTE: (**a**) Bode plot, and (**b**) phase angle.

EIS was conducted to further examine the effect of the MCSTE processing on the corrosion resistance of AZ31 alloy. Nyquist plots, equivalent circuit used to fit the EIS spectra and bode plots, and the Bode plot phase angles are presented in Figs. 11, 12 and Fig. 13, respectively. In addition, the electrical of the equivalent circuit are displayed in Fig. 12 while its parameters were tabulated in Table 5. The corrosive solution resistance is Rs, the polarization resistance is Rp, the oxide film capacitance is CPE, and the empirical exponent N, used to define CPE.

**Table 5:** Electrical parameters of the EIS equivalent circuit for AZ31 alloy.

Condition	Rs (Ω)	Rp (Ω)	CPE (F)	N
AA	848.68	174.25	6.028 × 10^–06^	0.8984
4P-30	603.67	362.98	1.4085 × 10^–03^	0.96115
8P-30	678.5	306.45	5.1936 × 10^–06^	0.91679
1P-40	650.6	256.14	5.6911 × 10^–06^	0.99318
2P-40	839.75	172.62	6.1624 × 10^–06^	0.99716

All AZ31 billets displayed single capacitive semicircles as shown in Nyquist plots (Fig. 11). The size of the capacitive semicircle radius is directly proportional to the corrosion resistance^75^. Furthermore, a slight inductive behavior can be seen for all the billets (Fig. 11) indicating surface dissolution due to the biodegradable nature of the alloy^76^. Similar ECAPed AZ31 alloy showed analogous inductive phenomenon^58^. From Nyquist plots it can be revealed that the AA billet displayed a smaller semicircle compared to all AZ31 MCSTE-processed billets. From Table 5, it can be revealed that the accumulation of plastic strain produced during 4P-30 processing resulted in a significant corrosion resistance improvement of 108% compared to the AA. Increasing the number of passes up to 8P-30 yielded an insignificant decrease of 15% in the corrosion resistance compared to 4P-30, while yielding a significant improvement of 76% compared to the AA counterpart. This insignificant reduction in corrosion resistance can be attributed to the increase in the dislocation density caused by processing for a high number of passes. On the other hand, processing through 1P-40 revealed a significant improvement of 47% in the corrosion resistance compared to the AA which can be attributed to grain refinement caused by processing. Processing through 2P-40 showed analogous corrosion resistance values to those of the AA counterpart. The Bode plots shown in Fig. 13a revealed similar impedance values for the AA and 2P-40 billets across all frequencies, while the rest of the billets showed lower impedance values. The AA and 2P-40 billets exhibited higher phase angles at lower frequencies while the 4P-30 billet experienced higher phase angles at intermediate and relatively high frequencies (Fig. 13b). Finally, at higher frequencies (greater than 104 Hz), the 2P-40 billet had the largest phase angles. Similar research on AZ31 processed through ECAP reported an average improvement in corrosion resistance of 70–80%^58^. The higher enhancement in corrosion resistance suggests that MCSTE can serve as a parallel technique to ECAP for various applications.

It is important to note that MCSTE processing, as a Mg-alloy SPD technique can result in the nucleation of many energetic crystalline defect sites. The alloy’s corrosion potential is then reduced to a more noble level because of these crystalline defects, as described in^73,76^. This occurs as a result of the defects encouraging the growth of protective magnesium oxide and hydroxide MgO/Mg(OH)_2_ layers^16,77^. Grain refinement, such as that caused by MCSTE processing, can further enhance the Mg(OH)_2_ protective coating, stimulating its growth and enhancing the alloy’s resistance to corrosion^77^.

**Fig. 14:**
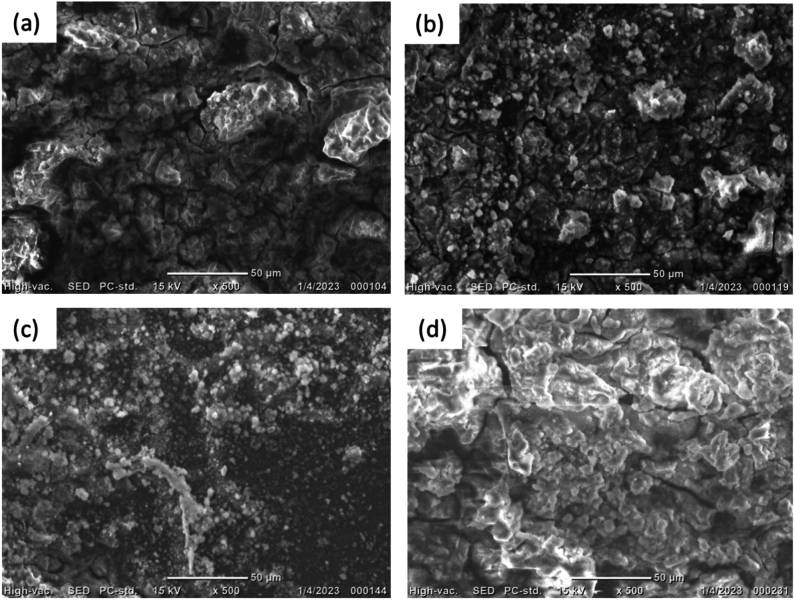
SEM micrographs of the AZ31 billets after corrosion testing. AA (**a**) 4P-30 (**b**), 8P-30 (**c**), and 2P-40 (**d**).

Figure 14 demonstrates the SEM micrographs of the processed and AA billets after corrosion tests. The predominant corrosion mechanism for the processed billets is intergranular corrosion. Moreover, increasing the number of passes increased the strength of the protective layer, which is evidenced by the proportional reduction in intergranular corrosion, compared to the AA condition, with increased number of passes (Fig. 14a). Processing through 4P-30° yielded a more stable and adhesive protective layer covering almost the entirety of AZ31’s surface, as shown in Fig. 14b. Increasing the number of passes up to 8P-30° yielded an almost pitting-free, thicker protective layer, as shown in Fig. 14c. However, processing through 2P-40° produced a less adherent protective layer than the 30°-die and very minor pitting was seen (Fig. 14d). As a result, the polarization resistance (Table 5) was observed to be lower when using the 40°-die than with the 30°-die. Accordingly, the stronger protective layer (Fig. 14) leads to enhance the corrosion resistance of AZ31 alloy which agrees with the findings of Y. Cubides et al.^16^. Furthermore, the refinement and redistribution of secondary phase particles (Mg_17_Al_12_) along the grain boundaries and triple junctions after SPD processes also plays an important role in improving the corrosion behavior of the Mg alloy which agrees with an earlier study^58^.

The immersion test was carried out using ringer lactate solution for 30 days (720 h) to investigate the degradation behavior of AZ31 alloy. Table 6 shows the weight loss in the billets over several points in time across the 30-day period. Weight loss measurements were made on a weekly basis using a sensitive scale. The weight before corrosion is denoted *Wb* and the weight after exposure to the corrosive medium for a specific number of days is denoted *Wa*. After 30 days the AA billet experienced a weight loss of 0.0043 gm. MCSTE processing via 4P-30 and 8P-30 displays reduced weight losses of 49% and 19%, respectively, compared to the AA condition. On the other hand, processing via a twist angle of 40° caused notable weight-loss decreases of 51% and 14% in the billets processed through 1P and 2P, respectively, compared to the AA condition. The immersion test findings are in agreement with the polarization curves and the EIS findings^78^. Comparable studies on ECAPed AZ31 showed decrease in weight loss by about 30% with respect to the unprocessed sample which indicates that MCSTE can be effective SPD technique akin to ECAP^73^.

**Table 6:** Weight loss after various times of immersion in ringer lactate solution of the billets before and after MCSTE processing.

Condition	Wb (gm)	Wa (Day 7) (gm)	Wa (Day 14) (gm)	Wa (Day 21) (gm)	Wa (Day 30) (gm)	Weight loss (gm)
AA	0.3126	0.3113	0.3103	0.3096	0.3083	0.0043
4P-30	0.3475	0.3464	0.3463	0.3457	0.3453	0.0022
8P-30	0.4060	0.4047	0.4038	0.4031	0.4025	0.0035
1P-40	0.3161	0.3158	0.3156	0.3152	0.3140	0.0021
2P-40	0.4037	0.4031	0.4021	0.4012	0.4000	0.0037

## Conclusions

The effects of MCSTE processing twist angles and number of passes on the crystallographic texture, microstructural evolution, electrochemical behavior, and mechanical properties of the biodegradable AZ31 Mg alloy have been examined in this article. After annealing, up to eight passes of MCSTE were accomplished using route C at 250°C. The subsequent conclusions are obtained:

1. MCSTE processing results in grain refinement of the billets and generation of twins in the deformed grains, and the twin density increased with increasing number of passes. XRD results confirm grain refinement and a uniform distribution of finer β-Mg_17_Al_12_ precipitation phases with increased number of passes. However, the high induced strain via the 40° twisting die results in increased localized nucleation sites for the phase nucleation.
2. MCSTE processing via 30° twisting die shows significant grain refinement of about 66% for the 4P billet and 72% for the 8P billet coupled with a notable increase in fraction of LAGBs.
3. Processing through 1P and 2P using the 40º twisting channel displayed a significant refinement of the grain size of 73.1% and 75.6%, respectively compared to the AA counterpart.
4. MCSTE processing via 30° twist angle results in significant increase in compressive YS (43%), UTS (31%) and ductility (11%) with increasing the number of passes up to 8P compared to the AA billets.
5. MSCTE 4P-30º and 8P-30º processing improve corrosion rate by about 88% and 76%, respectively, compared to the AA billet.
6. MSCTE 4P-30 processing shows better corrosion properties with corrosion resistance being improved by about 108% with respect to AA. However, 8P-30 exhibits a decreased corrosion resistance of about 15% compared to 4P-30 due to the increased density of dislocations at higher number of passes.
7. The exceptional balance of mechanical performance and corrosion resistance achieved by MCSTE processing via 4P-30 shows that MCSTE-processed AZ31 is a viable method for developing biodegradable applications.

## Data Availability

"The datasets generated during and/or analyzed during the current study are available upon request".
